# Evaluation of Current and New Biomarkers in Severe Preeclampsia: A Microarray Approach Reveals the *VSIG4* Gene as a Potential Blood Biomarker

**DOI:** 10.1371/journal.pone.0082638

**Published:** 2013-12-09

**Authors:** Julien Textoris, Delphine Ivorra, Amira Ben Amara, Florence Sabatier, Jean-Pierre Ménard, Hélène Heckenroth, Florence Bretelle, Jean-Louis Mege

**Affiliations:** 1 Aix-Marseille Université, Unité de Recherche sur les Maladies Infectieuses Tropicales et Emergentes, CNRS UMR 7278, INSERM U1095, Marseille, France; 2 Aix-Marseille Université, Department of Gynecology and Obstetrics, Hôpital Nord, Marseille, France; 3 Aix-Marseille Université, Laboratoire de Physiopathologie de l'Endothélium, Unité mixte de recherche, S 608 INSERM, Marseille, France; 4 Aix-Marseille Université, Department of Gynecology and Obstetrics, Hôpital de la Conception, Marseille, France; VU University Medical Center, Netherlands

## Abstract

Preeclampsia is a placental disease characterized by hypertension and proteinuria in pregnant women, and it is associated with a high maternal and neonatal morbidity. However, circulating biomarkers that are able to predict the prognosis of preeclampsia are lacking. Thirty-eight women were included in the current study. They consisted of 19 patients with preeclampsia (13 with severe preeclampsia and 6 with non-severe preeclampsia) and 19 gestational age-matched women with normal pregnancies as controls. We measured circulating factors that are associated with the coagulation pathway (including fibrinogen, fibronectin, factor VIII, antithrombin, protein S and protein C), endothelial activation (such as soluble endoglin and CD146), and the release of total and platelet-derived microparticles. These markers enabled us to discriminate the preeclampsia condition from a normal pregnancy but were not sufficient to distinguish severe from non-severe preeclampsia. We then used a microarray to study the transcriptional signature of blood samples. Preeclampsia patients exhibited a specific transcriptional program distinct from that of the control group of women. Interestingly, we also identified a severity-related transcriptional signature. Functional annotation of the upmodulated signature in severe preeclampsia highlighted two main functions related to “ribosome” and “complement”. Finally, we identified 8 genes that were specifically upmodulated in severe preeclampsia compared with non-severe preeclampsia and the normotensive controls. Among these genes, we identified *VSIG4* as a potential diagnostic marker of severe preeclampsia. The determination of this gene may improve the prognostic assessment of severe preeclampsia.

## Introduction

Preeclampsia (PE) is a placental disease characterized by the onset of hypertension and proteinuria after 20 weeks of gestation [1]. PE occurs in 2-8% of pregnancies [2] and is a leading cause of feto-maternal morbidity and mortality [1]. The clinical presentations of PE are heterogeneous, which makes effective treatment difficult. Two nonexclusive theories account for the pathophysiology of PE. The first emphasizes the vascular aspect of the pathophysiology, including placental hypoxia, enhanced platelet aggregation and endothelial dysfunction. Endothelial dysfunction is believed to persist after delivery, thus explaining maternal and neonatal complications. The second theory focuses on immunological dysfunction, whereby fetal tissue is involved in the onset and duration of the clinical presentation [3,4]. It is likely that PE encompasses a combination of genetic, immune and biological aspects, making it a multifactorial disease. This characteristic explains the difficulty in identifying specific biomarkers and designing efficient therapies. Indeed, numerous circulating factors have been investigated, but none have been identified as consistent biomarkers [5–8]. After promising initial results, recent advances have shown that angiogenic factors, such as soluble endoglin (sEndoglin), soluble vascular endothelial growth factor receptor (sVEGF-R, also known as sFLT1) and placental growth factor (PlGF) are not predictive of early PE [9–11]. Microparticles have been associated with recurrent miscarriages and PE and have been evaluated for their diagnostic properties [12,13]. An approach combining the clinical and biological features of PE has a better predictive value, likely reflecting the multifactorial origin of PE [14].

The development of new high-throughput methods enables the identification of novel biomarkers and increases the probability of their use in clinical practice [15]. Many studies have applied microarray analyses of placental biopsies to determine potential biomarkers of PE [16]. The work has revealed gene expression differences in placentas from PE women compared with normotensive (NT) women [17–21]. These changes have a relative predictive value because dysregulated gene expression in the early placenta (samples were obtained from the chorionic villous) is observed 6 months before the development of PE [19]. The functional annotation of the modulated genes in PE shows an enrichment in inflammatory immune and metabolic categories [17]. Some of the gene products have been identified as potential biomarkers of PE, including leptin [17], endoglin pathway genes [22], platelet-derived growth factor (PDGF) [21], VEGF and its soluble receptor [23], and metalloprotease (MMP)-12 [24]. However, the invasive sampling approach precludes its routine use. Three studies have focused on the analysis of the transcriptome in blood samples taken from a limited number of patients with PE. These studies demonstrated the modulation of some genes, but only a few were shared, confirming that larger cohorts of patients are required to verify these results [25,26].

In the current study, we showed that circulating factors associated with the coagulation pathway, endothelial activation and microparticle release were modulated in PE compared with NT controls, but these factors did not discriminate severe and non-severe PE. We therefore used a microarray approach to further distinguish severe and non-severe PE. Overall, the pattern of gene expression was distinct between PE and control women, and the functional annotation of the upmodulated signature in severe PE highlighted functions related to “ribosome” and “complement”. A subanalysis showed that 8 genes, including the *VSIG4* gene, were specifically upmodulated in severe PE. This gene may serve as a potential diagnostic marker of severe PE and improve the prognostic assessment of severe PE.

## Materials and Methods

### Patients

The study was approved by the Ethics Committee "CPP Sud Méditerranée 1" (n° 2010-A00633-36, on 05/11/2010). Written informed consent was obtained from each pregnant woman. Thirty-eight women were included in the study: 19 patients with PE, including 6 women with non-severe PE and 13 women with severe PE, and 19 NT women selected according to age, weight, smoking status, race, gestational age at the inclusion and blood pH (Table **1**). NT women had no history of medical illness or use of medication and received routing prenatal care. The diagnosis of PE was based on a blood pressure of ≥ 140/90 mmHg taken twice, uricemia, and a positive proteinuria either on urine stick or an urine sample, occurring after 20 gestational weeks in previously normotensive women (Table **2**). Following the ACOG definition [27], severe PE was defined by the presence of one or more of the following conditions: a blood pressure higher than 160/110 mmHg, a proteinuria higher than 5000 mg/24h, a multisystem disorder, maternal cerebral symptoms (seizures, stroke) or an intrauterine growth restriction below the 3rd percentile. Women with multiple gestations, fetal congenital malformations/chromosomal abnormalities, recent infection, antiphospholipid antibodies, trauma, drug or alcohol abuse during pregnancy, preexisting hypertension, thrombophilia with a history of PE or receiving anticoagulant or anti-aggregation therapy were excluded from the study. 

**Table 1:** Clinical characteristics of patients.

	**Controls (n = 19)**	**Non severe PE (n = 6**)	**Severe PE (n = 13)**	***p***
Maternal age (years)	28 [26-32]	36 [33-37]	30 [27-34]	0.17
Weight (kg)	70 [64-83]	77 [72-81]	78 [66-86]	0.60
Smoking	1 (5)	1 (17)	1 (8)	1
Previous PE	0	0	3 (23)	0.52
Caucasian race	9 (47)	4 (67)	9 (69)	1
Gestity / Parity	G2/P1 [1/0 - 3/2]	G3/P1 [2/0 - 4/2]		1
Gestational age at inclusion	36 [33-37]	34 [32-36]	34 [31-35]	0.79
Gestational age at birth	40 [39-40]**	36 [35-38]	34 [31-36]	0.12
Birth weight (g)	3240 [2990-3620]**	1805 [1495-1930]	1900 [1060-2120]	0.92
Blood pH at birth	7.32 [7.25-7.34]	7.29 [7.21-7.30]	7.33 [7.28-7.36]	0.12

The values were expressed as median and interquartile range (in brackets) or absolute count and percentages (in parentheses). The *p* values correspond to the comparison between non-severe and severe PE. The asterisks correspond to the comparison between controls and PE patients independently of the severity of the disease. *p < 0.01; **p < 0.001.

**Table 2:** Biological characteristics of patients.

	**Controls (n = 19)**	**Non severe PE (n = 6)**	**Severe PE (n = 13)**	***p*-value**
Proteinuria (g/24h)	0**	0.38 [0.20-0.38]	1.00 [0.53-3.00]	0.02
Uricemia (µmol/L)	277 [196-292]*	365 [289-384]	336 [286-382]	0.78
Platelets (10^9^/L)	236 [212-259]	209 [181-258]	259 [229-347]	0.22
Neutrophils (10^9^/L)	8.2 [7.0-10.3]	6.7 [5.3-7.0]	9.2 [5.3-13.3]	0.10
Lymphocytes (10^9^/L)	2.2 [1.6-2.4]	2.2 [1.7-2.5]	2.1 [1.3-2.9]	0.86
Monocytes (10^9^/L)	0.6 [0.6-0.8]	0.5 [0.4-0.5]	0.6 [0.5-0.7]	0.28
ASAT (UI/L)	NA	24 [22-27]	21 [19-24]	0.15
ALAT (UI/L)	NA	17 [11-21]	16 [14-19]	0.90
LDH (UI/L)	NA	457 [310-506]	386 [204-540]	1

The values were expressed as median and interquartile range (in brackets). As inclusion criteria for proteinuria was based on a positive urinary stick or an urine sample, one patient from the non-severe group displayed a low value on the 24h collection time proteinuria (0.13g/24h). The asterisks correspond to the comparison between controls and PE independently of the severity of the disease. The *p* values correspond to the comparison between non-severe and severe PE. *p < 0.01; **p < 0.001. ASAT: aspartate amino transferase; ALAT: alanine amino transferase ; LDH: lactate dehydrogenase. NA: not available.

### Circulating angiogenic factors

The angiogenic factors sFLT1, sEndoglin and PlGF were measured in citrate plasma using commercially available ELISA kits (R&D Systems, Lille, France). Soluble CD146 (sCD146) was measured using an ELISA kit from Biocytex (Marseille, France).

### Microparticles

Total (Annexin V^+^) and platelet-derived (CD41^+^) microparticles were measured by flow cytometry as previously described [28]. Briefly, whole blood was drawn into sodium citrated tubes and centrifuged within 2 hours of collection at 1,500 x *g* for 15 min. Residual platelets were removed by centrifugation at 13,000 x *g* for 2 min, and plasma containing microparticles was stored at -80°C until analysis. After thawing, 30 µL of the plasma samples was incubated for 30 min with Annexin V-FITC and CD41-PE or isotype controls (IgG1-PE) obtained from Beckman Coulter (Villepinte, France). Microparticles were measured on an FC500 flow cytometer from Beckman Coulter using calibrated Megamix beads (Biocytex). Flow Count Beads (Beckman Coulter) were added to each sample to express the microparticle counts as absolute numbers.

### Microarrays

Venous blood samples (2.5 mL) were collected at the time of study inclusion in PAXgene Blood RNA tubes (PreAnalytiX, Hombrechtikon, Switzerland) and were kept at room temperature for 2 hours before freezing. RNA was extracted using the PAXgene Blood RNA kit (Qiagen, Courtaboeuf, France) with a DNase I step included to eliminate DNA contaminants according to the manufacturer’s recommendations. The quantity and quality of the RNA were assessed using a Nanodrop (Thermo Science, Orsay, France) and a 2100 Bioanalyseur (Agilent Technologies, Massy, France), respectively. The microarray study was performed using microarray chips that included 45,000 probes (1 microarray for each patient, 4X44K Whole Human Genome microarray G4112F) and the One Color Microarray Based Gene Expression Analysis based on the Agilent Technologies procedures recently described [29]. In brief, 400 ng of RNA was labeled with cyanine-3 CTP using the quick amp labeling kit (Agilent Technologies, Massy, France). The hybridization was performed for 17 hours at 65°C using the gene expression hybridization kit (Agilent Technologies, Massy, France). The microarray slides were scanned with a G2505C DNA Microarray scanner at a resolution of 5 µm. The scanned images were analyzed using the Feature Extraction 10.5.1 software. 

### Microarray analysis

Analysis of the microarray data was performed, and the figures were created using the R (v.2.13) and Bioconductor software suite as recently described [30]. The raw data were filtered and normalized using the *Agi4x44PreProcess* library. Two microarrays were discarded because they did not pass the quality control check. Unsupervised and supervised analyses were performed using hierarchical clustering, principal component analysis (*made4* library) [31] and the Linear Models for Microarray Analysis (*limma* library) [32]. We considered genes to be differentially expressed when the adjusted *p* value was below 0.05, and the absolute fold change (FC) was higher than 1.5. The average linkage was based on the Pearson correlation distance. Functional enrichment analysis was performed on selected genes with the DAVID bioinformatics tool using Gene Ontology (GO) pathways. Keywords were selected when the Benjamini-Hochberg-corrected *p*-value for enrichment was less than 0.01. The data were generated in compliance with the MIAME guidelines and were deposited in the National Center for Biotechnology Information's Gene Expression Omnibus (accession number: GSE48424).

### Quantitative real-time RT-PCR

Potential severity-associated genes identified by the microarray were selected and validated by quantitative real-time RT-PCR (qRT-PCR) as previously described [29]. Reverse transcription of 100 ng of RNA was performed with the M-MLV-RT kit (Invitrogen, Life Technologies, Saint-Aubin, France). cDNA was obtained using oligo(dT) primers and the M-MLV reverse transcriptase, and quantitative PCR was performed using the SYBR Green Fast Master Mix (Roche Diagnostics, Meylan, France) and an ABI7900 Fast Real-Time PCR machine (Life Technologies). The primers were designed using Primer3 [33] and their sequences are presented in Table **S1**. The results were normalized using the housekeeping gene β-actin and were expressed as FC = 2^-∆∆Ct^, where ∆∆Ct = (Ct_Target_-Ct_Actin_)_PE_ - (Ct_Target_-Ct_Actin_)_NT control_ as previously described [34].

### Statistical analysis

Quantitative parameters were expressed as the median and inter-quartile range. Qualitative parameters were expressed as absolute counts and percentages. Differences between groups were assessed with the nonparametric Wilcoxon rank sum test. Differences were considered as significant when the *p* value was lower than 0.05.

## Results

### Characterization of PE patients

We included 19 PE women matched with 19 NT women in the study. The diagnosis of PE was based on changes in blood pressure, proteinuria and increased uricemia (Table **2**). We first analyzed the clinical and biological features known to be associated with PE. The maternal age, weight and gestational age at inclusion were similar between the two groups of patients. As expected, the gestational age at birth and the birth weight were significantly (*p* < 0.001) lower in PE women than in NT controls (Table **1**). Among the biological parameters, we did not find any differences in circulating leukocytes and platelets (Table **2**). The PE patients were divided into 2 subgroups according to the severity of the disease (6 and 13 patients with non-severe and severe PE, respectively). A comparison of the two groups revealed that proteinuria was higher in the severe PE than in the non-severe PE patients (1.00 [0.53-3.00] g/24h *vs.* 0.38 [0.20-0.38]; *p* = 0.02), but that other clinical (Table **1**) and biological (Table **2**) parameters were similar between these patients. 

### Measurement of potential biomarkers in PE patients

We measured biological parameters associated with either the vascular or inflammatory hypothesis and biomarkers previously identified in placentas from PE patients in circulating blood. The circulating levels or the activity of molecules involved in coagulation or fibrinolysis, such as fibrinogen, fibronectin, factor VIII, antithrombin, protein S and protein C, were similar in the PE patients and NT controls. The severity of PE did not affect the levels or activity of these molecules with the exception of protein C, which was significantly higher (*p* < 0.02) in severe PE than in non-severe PE but without clinical significance (111 [106-116] % in severe PE vs. 93 [81-99] % in non-severe PE). We also found that potential biomarkers, such as sEndoglin and sCD146, were not significantly modulated in PE patients. In contrast, PlGF was significantly downmodulated in PE patients (63 [50-88] pg/mL vs. 225 [104-282]; *p* < 0.001), independently of the degree of severity. Finally, microparticle levels (total and platelet-derived microparticles) were increased in PE patients compared with NT controls, but the levels were similar between the severe and non-severe PE patients (Table **3**). Taken together, our results demonstrate that PlGF is the only diagnostic biomarker that is significantly modulated between PE patients and NT controls. However, none of these biomarkers is able to assess the severity of PE. 

**Table 3:** Potential circulating biomarkers of PE patients.

	**NT controls**	**non-severe PE**	**severe PE**	***p* value**
Fibrinogen (g/L)	4.82 [4.21-5.86]	4.94 [4.87-5.12]	4.42 [4.27-5.34]	0.52
Fibronectin (g/L)	NA	0.54 [0.36-0.73]	0.61 [0.59-0.71]	0.46
Factor VIII activity (%)	2.0 [1.6-2.2]	2.4 [1.6-3.0]	2.4 [1.8-2.5]	1
Antithrombin (%)	94 [88-99]	82 [81-86]	91 [86-93]	0.27
Protein S (%)	48 [40-63]	66 [60-69]	52 [42-62]	0.12
Protein C (%)	98 [90-123]	93 [81-99]	111 [106-116]	0.02
sEndoglin (ng/mL)	4.8 [3.7-7.9]*	25.5 [14.0-31.2]	20.8 [11.2-30.8]	0.79
sCD146 (ng/mL)	159 [154-191]	182 [177-187]	149 [140-169]	0.25
sFLT1 (pg/mL)	2815 [1640-21980]*	13010 [8633-18257]	12367 [10871-13280]	0.85
PlGF (pg/mL)	225 [104-282]**	61 [54-73]	75 [44-91]	0.52
Total MP (number/µL)	595 [360-755]*	979 [599-1205]	1316 [812-1415]	0.29
Platelet-derived MP (number/µL)	515 [267-761]*	706 [474-1152]	947 [863-1352]	0.19

The values were expressed as median and interquartile range (in brackets). The *p* values correspond to the comparison between non-severe and severe PE. The asterisks correspond to the comparison between controls and PE independently of the severity of the disease. *p < 0.05; **p < 0.001. MP: microparticles; sFLT1: soluble vascular endothelial growth factor receptor; PlGF: Placental Growth Factor.

### Transcriptional signature of PE patients

**Figure 1:**
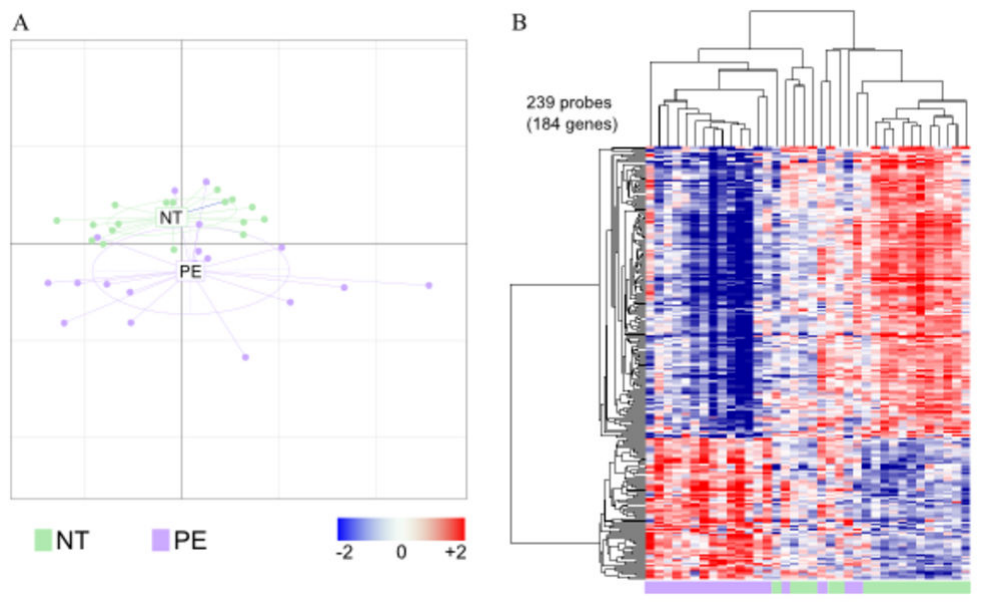
Preeclampsia related transcriptional signature. RNA was extracted from whole blood and microarrays were performed. **A**, The impact of the PE disease was analyzed by principal component analysis. The distance along each axis represents the degree of variance in gene expression. **B**, Supervised analysis using linear models identified 239 probes (184 genes) modulated in preeclampsia (PE, in purple) patients compared with normotensive (NT, in green) patients. These modulated probes are represented as a heatmap with probes in rows and samples in columns. Dendrograms show the results of the hierarchical clustering of the probes (left) and samples (top). Gene expression is represented by a color gradient ranging from blue (downmodulated genes) to red (upmodulated genes).

Because the use of single biomarkers was not sufficient to assess the degree of PE severity, we used a whole-genome microarray approach to define the peripheral blood transcriptional signature of PE. The principal component analysis showed that the overall gene expression of PE women and control patients was organized in two different groups. Note that the variance in gene expression appeared to be higher in the PE group than the control group (Figure **1A**), suggesting a relative heterogeneity of PE patients. Supervised analysis using the SAM algorithm identified 239 probes (184 genes) that were modulated in PE patients compared with control women (Figure **1B**). Among the probes modulated in PE, 161 probes (150 genes) were downmodulated and 78 (45 genes) were upmodulated (Table **S2**). The functional annotation of the upmodulated genes identified specific enriched functions such as "regulation of molecular function", “cell communication”, “intracellular signaling cascade”, “cell cycle”, “cell differentiation” and “gene expression”. Many genes were also annotated with keywords linked to key cellular functions, such as “Regulation of immune process”, “membrane organization”, “hemopoiesis”, and “cell death” (Table **4**). Taken together, these results show that PE patients exhibit a specific transcriptional program compared with NT controls. 

**Table 4:** Functional annotation of upmodulated genes in PE.

**GO ID**	**GO term**	**Number of associated genes**	***p* value**
0065009	regulation of molecular function	19	1.7 × 10^-3^
0007154	cell communication	59	3.2 × 10^-3^
0007049	cell cycle	17	2.8 × 10^-3^
0030154	cell differentiation	21	5.9 × 10^-2^
0030029	actin-filament based process	8	8.3 × 10^-3^
0050896	response to stimulus	29	6.6 × 10^-1^
0042060	wound healing	5	2.1 × 10^-2^
0009893	positive regulation of metabolic process	25	1.1 × 10^-8^
0008219	cell death	17	2.2 × 10^-4^
0006950	response to stress	20	1.3 × 10^-1^
0016044	membrane organization	12	8.4 × 10^-4^
0007242	intracellular signalling cascade	35	1.4 × 10^-6^
0007165	signal transduction	56	1.7 × 10^-3^
0002682	regulation of immune system process	7	4.5 × 10^-2^
0030097	hemopoiesis	8	2.0 × 10^-3^
0010467	gene expression	60	6.5 × 10^-9^

The genes that were specifically upmodulated in PE compared to NT controls were annotated using GO database. The number of modulated genes associated with a particular term is indicated. The *p* value was obtained after Benjamini-Hochberg correction.

### Transcriptional signature of severe PE

**Figure 2:**
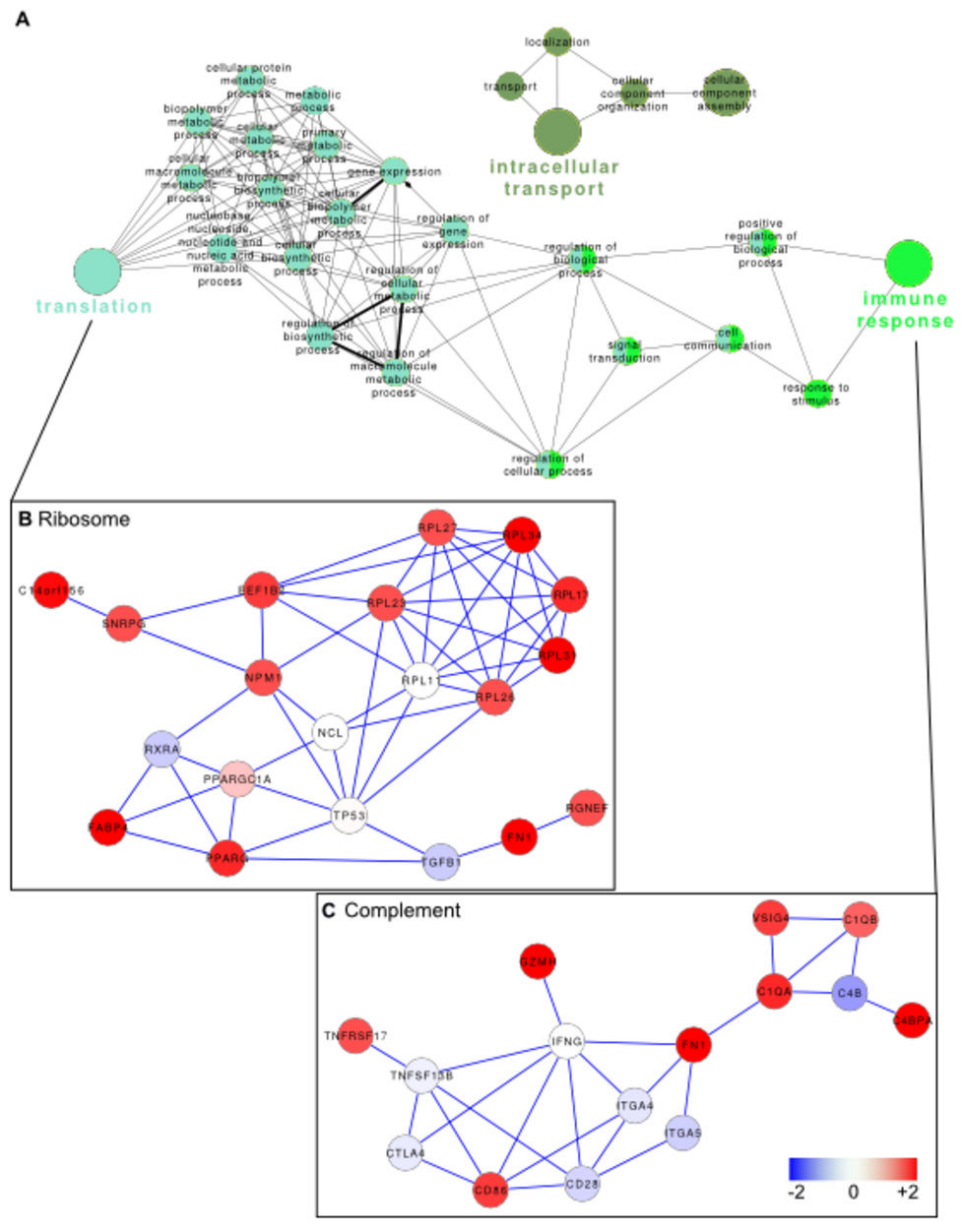
Functional annotation of genes modulated in severe PE. The genes that were modulated in severe PE compared with non-severe PE were analyzed using GO terms in Cytoscape. **A**, The network represents the relationship between the significant GO terms that were associated with the identified genes. **B**, An interactome map was built from the genes associated with the terms of the “ribosome” cluster, and gene expression in severe PE is color-coded from blue (downmodulated) to red (upmodulated). **C**, An interactome map was built from the genes associated with the terms of the “complement” cluster, and gene expression in severe PE is color-coded from blue (downmodulated) to red (upmodulated).

As the variability of gene expression patterns was higher in PE patients than NT controls, we analyzed the modulation of gene expression according to PE severity to identify severity-associated biomarkers. In a supervised analysis comparing severe and non-severe PE, we found 116 probes (69 genes) that were upmodulated in severe PE (Table **S3**). Functional annotation of this signature highlighted three main functions related to “intracellular transport”, “translation” and “immune response” (Figure **2A**). The interactome map of the modulated genes associated with the “translation” cluster showed that the upregulation of many genes encoding proteins of the large ribosome subunit (*RPL17, RPL23, RPL26, RPL27, RPL31* and *RPL34*) and involved in ribosome biogenesis (*SNRPG, NPM1, EEF1B2*) were up-modulated in women with severe PE (Figure **2B**). The “immune response” cluster included four genes related to the complement system (*C1QA, C1QB, C4BPA, VSIG4*) (Figure **2C**).

**Figure 3:**
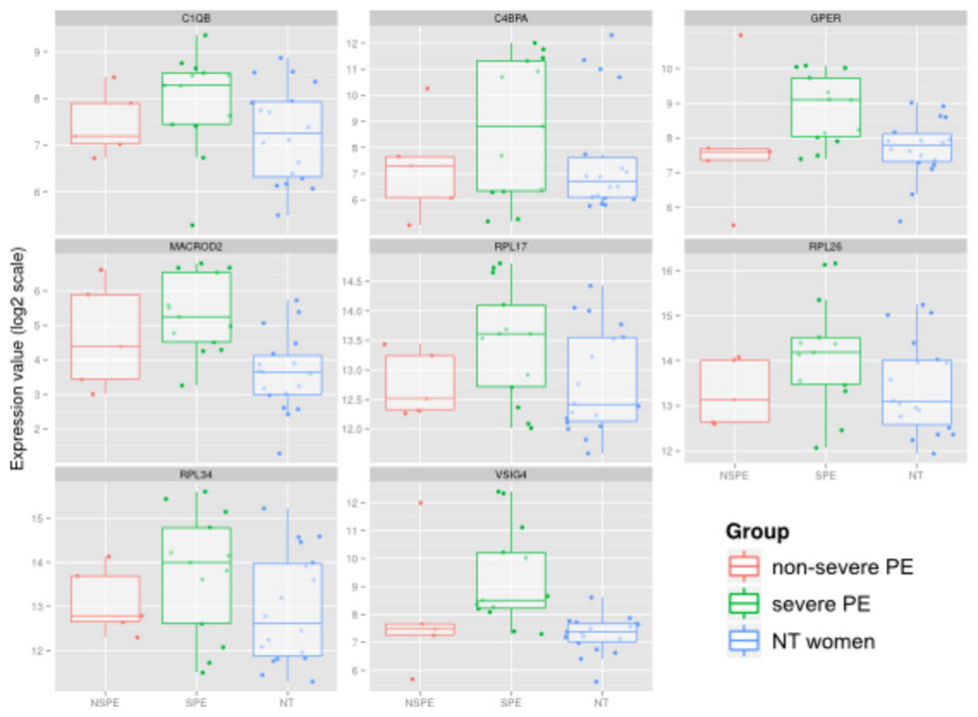
Expression of potential biomarkers of severity of PE. Eight genes were selected as potential biomarkers of the severity of PE. Their expression in the microarray is represented as box and whiskers, according to the disease status and severity of PE: normotensive (NT) patient (blue), non-severe PE (red) and severe PE (green). Individual results are shown with box and whisker representation.

We then selected 8 genes that were specifically upmodulated in severe PE compared with NT controls but that were not modulated in non-severe PE (Figure **3**). Among these genes, *C1QB*, *C4BPA* and *VSIG4* genes were related to the complement system and *RPL17*, *RPL26* and *RPL34* to the ribosome pathway. Interestingly, we also identified the gene encoding the estrogen receptor (*GPER*) in this signature. The specific modulation of *RPL26, RPL34, MACROD2* and *VSIG4* genes was also confirmed by qRT-PCR (Table **5**). The severity ratio was higher for *VISG4* (severe/non-severe ratio = 8.8) than other genes. Taken together, our approach identified potential diagnostic biomarkers of PE severity.

**Table 5:** Analysis of severity-associated biomarkers by qRT-PCR.

	Microarray ratio		qRT-PCR ratio	
	non-severe	severe	non-severe	severe
RPL26	1.0	2.1	2.6	13.2
RPL34	1.1	2.6	1.9	6.2
MACROD2	1.6	3.1	3.7	5.8
VSIG4	1.1	2.2	0.9	7.9

The selected biomarkers identified in microarray were analyzed by qRT-PCR. The results were expressed relative to NT controls. The Spearman correlation coefficient was 0.6

## Discussion

The objective of our study was to identify new circulating biomarkers of PE severity. To date, the most relevant serum biomarker identifying severe PE is the variation in plasma sEndoglin levels during the pregnancy trimesters [35]. A ratio between soluble FLT1 and PlGF in the first trimester may predict an evolution toward PE, but this ratio is not related to severity [36].

We chose to study circulating cells because this method is non-invasive and may be easily applied to the general population. First, we selected different biomarker candidates from the literature [9,11]. Most of them were modulated in PE independently of the degree of severity of the disease. The measurement of circulating microparticles was also evaluated. Microparticles are associated with thrombotic conditions, and their increase has been documented in women with recurrent miscarriage, PE, intrauterine growth retardation and gestational hypotension [37,38]. We found that total microparticles and platelet-derived microparticles, known to be highly thrombogenic, were clearly increased in PE compared with NT women. Again, their measurement could not discriminate severe PE from non-severe PE, thereby excluding microparticles as a potential marker of PE severity. 

Microarray analysis may be an excellent technology to study a systemic disorder such as PE. Indeed, it allows the large-scale screening of biomarkers, and a peripheral transcriptional signature of PE has been reported in a limited number of patients [25]. A transcriptional signature associated with PE severity has also been described in a small cohort of patients [26]. Nevertheless, the comparison of these two microarray analyses is difficult because different microarrays or various variable filtering or statistical methods were used. In our study, we included a larger cohort of patients and matched controls. Despite a relative heterogeneity in the PE patients, we identified a disease-related signature in which 184 genes were significantly modulated, and the functional categories were related to cell proliferation, differentiation and apoptosis. Genes from the cell proliferation and apoptosis categories were also found in PE patients from Rajakumar's study [25]. Importantly, a transcriptional signature of severe PE was also identified in the current study. This signature included the overexpression of the ribosomal genes *RPL26*, *RPL34*, *RPS15A* and *RPS26*, along with genes encoding proteins involved in ribosome biogenesis, suggesting that severe PE is characterized by a selective modulation of the translational machinery. It also included *GPER* that encodes an oestrogen receptor. The more interesting effect of GPER in the context of PE is its effect on the cardiovascular system and regulation of blood pressure [39]. PE has been recently reported to increase the long-term risk of cardiovascular disease with increased maternal mortality, and this risk factor may be related to estrogen levels [40,41]. 

We also found that some genes belonging to the complement pathway were upmodulated in severe PE compared with non-severe PE. There is already accumulating evidence for a role of complement in the pathogenesis of several complications of pregnancy, including PE, intrauterine fetal death and recurrent spontaneous abortion [42,43]. The activation of complement is necessary to maintain immune homeostasis during pregnancy, but excessive or inappropriate activation of complement contributes to the pathogenesis of PE [44]. Our data suggested that molecules of the classical complement pathway were altered in PE, especially C1q and C4bp. In addition, the alteration of complement may be involved in renal lesions of PE as suggested by the aggravation of these lesions in genetic defects of complement regulatory proteins and the deposition of C1q in glomerular capillaries [45]. The deposition of C4BP along the glomerular capillary wall has also been found in PE patients [46].

Interestingly, the *VSIG4* gene was specifically upmodulated in severe PE. This result is consistent with that obtained by Sun et al. who found that the *VSIG4* gene was slightly overexpressed in peripheral blood mononuclear cells from patients with PE [26]. VSIG4 is a member of the B7 family that is only present on resident placental macrophages [47] and is involved in T-cell anergy [48]. VSIG4 belongs to the complement pathway due to its ability to bind C3b, C3i [49] and C1q (interactome databases). Because the *VSIG4* gene is the only gene found to be modulated in PE in the transcriptional studies published to date [25,26], it represents a superior candidate biomarker to assess PE severity. Given the large variation in expression value and the overlap between the three groups in our dataset, this biomarker requires validation in an independent cohort of patients. Finally, as PAXgene tubes were used in this study, the transcriptome we analyzed comes from heterogeneous origins (mainly leukocytes). Therefore, despite interesting pathophysiological hypothesis were raised, they should be confirmed by further studies.

In the present study, we evaluated a large panel of PE biomarkers. We confirmed the diagnostic value of most of them, but they were unable to predict the severity of the disease. We completed the analysis with a transcriptional study that identified several circulating biomarkers of PE severity. One of them, *VSIG4*, is a potential candidate that may help physicians to manage PE patients.

## Supporting Information

Table S1 **Nucleotide sequences of oligonucleotide primers.** Oligonucleotide primers used for qRT-PCR validation.
(DOC)

Table S2 **Genes modulated in pre-eclampsia (severe or non severe, versus control women).** List of the modulated genes in pre-eclampsia with probe IDs, gene symbols and fold-changes.
(XLS)

Table S3 **Genes modulated in severe pre-eclampsia versus non-severe pre-eclampsia.** List of the modulated genes in severe pre-eclampsia with probe IDs, gene symbols and fold-changes. both methods was 0.6.
(XLS)
