# Supplementary material for: Evaluation of Current and New Biomarkers in Severe Preeclampsia: A Microarray Approach Reveals the VSIG4 Gene as a Potential Blood Biomarker
Source: PLoS One. 2013 Dec 9;8(12):e82638. doi: 10.1371/journal.pone.0082638 (PMC3859491; doi:10.1371/journal.pone.0082638)
Supplement: Table S1 — Nucleotide sequences of oligonucleotide primers. Oligonucleotide primers used for qRT-PCR validation. (DOC) [file pone.0082638.s001.doc]

**Table S1. Nucleotide sequences of oligonucleotide primers**

| **Target gene** | **Forward** | **Reverse** |
| --- | --- | --- |
| RPL26 | 5'- GATTATGTCTTCCCCTCTTTCC -3' | 5'- CAGTTGTGCCATTAGCCTTTTC -3' |
| RPL34 | 5'- CCGAACCCCTGGTAATAGAA -3' | 5'- CTTGTGCCTTCAACACTTTCAC -3' |
| MACROD2 | 5'-GACAGAGGAGGCAGGATAGAAA -3' | 5'-GTTAGGAAGGAAGAGACCCAGA -3' |
| VSIG4 | 5'- CTCCTTGTGCTGTATGGTGGT -3 | 5'- ATCAGAGTAGTTGTTGCCCAGA -3' |
| ACTINB | 5'- GGAAATCGTGCGTGACATTA -3' | 5'- AGGAAGGAAGGCTGGAAGAG -3' |
